# Inferring within‐flock transmission dynamics of highly pathogenic avian influenza H5N8 virus in France, 2020

**DOI:** 10.1111/tbed.14202

**Published:** 2021-07-27

**Authors:** Timothée Vergne, Simon Gubbins, Claire Guinat, Billy Bauzile, Mattias Delpont, Debapriyo Chakraborty, Hugo Gruson, Benjamin Roche, Mathieu Andraud, Mathilde Paul, Jean‐Luc Guérin

**Affiliations:** ^1^ IHAP University of Toulouse, INRAE, ENVT Toulouse France; ^2^ The Pirbright Institute Pirbright Surrey UK; ^3^ Department of Biosystems Science and Engineering ETH Zürich Basel Switzerland; ^4^ Swiss Institute of Bioinformatics (SIB) Lausanne Switzerland; ^5^ MIVEGEC Université de Montpellier, IRD, CNRS Montpellier France; ^6^ IRD Sorbonne Université Bondy France; ^7^ Departamento de Etología, Fauna Silvestre y Animales de Laboratorio Facultad de Medicina Veterinaria y Zootecnia Universidad Nacional Autónoma de México (UNAM) Ciudad de México México; ^8^ ANSES Ploufragan‐Plouzané‐Niort Laboratory Epidemiology Health and Welfare Research Unit Ploufragan France

**Keywords:** avian flu, beta, duck, inference, mechanistic model, mortality, R0, spread

## Abstract

Following the emergence of highly pathogenic avian influenza (H5N8) in France in early December 2020, we used duck mortality data from the index farm to investigate within‐flock transmission dynamics. A stochastic epidemic model was fitted to the daily mortality data and model parameters were estimated using an approximate Bayesian computation sequential Monte Carlo (ABC‐SMC) algorithm. The model predicted that the first bird in the flock was infected 5 days (95% credible interval, CI: 3–6) prior to the day of suspicion and that the transmission rate was 4.1 new infections per day (95% CI: 2.8–5.8). On average, ducks became infectious 4.1 h (95% CI: 0.7–9.1) after infection and remained infectious for 4.3 days (95% CI: 2.8–5.7). The model also predicted that 34% (50% prediction interval: 8%–76%) of birds would already be infectious by the day of suspicion, emphasizing the substantial latent threat this virus could pose to other poultry farms and to neighbouring wild birds. This study illustrates how mechanistic models can help provide rapid relevant insights that contribute to the management of infectious disease outbreaks of farmed animals. These methods can be applied to future outbreaks and the resulting parameter estimates made available to veterinary services within a few hours.

## 1 INTRODUCTION

On 21 October 2020, the detection of highly pathogenic avian influenza (HPAI) H5N8 virus in two mute swans in the Netherlands raised concerns about the re‐emergence and further spread of this subtype, which has caused regular epidemics in the past few years. As of 19 November 2020, 302 HPAI (H5) detections had been reported in Europe, mainly in wild birds (Adlhoch et al., 2020). Since then HPAI (H5N8) outbreaks have been reported in poultry in various countries, with the highest number of outbreaks having been notified in France, Germany and Poland (IZSVe, 2020).

On 5 December 2020, rapidly increasing mortality was reported in a breeding mule duck farm in southwest France. In the hours that followed, the French national reference laboratory for avian influenza confirmed the presence of HPAI H5N8 virus subtype (clade 2.3.4.4.b). This was the first reported outbreak of HPAI in a French poultry farm since the devastating epidemic of HPAI (H5N8) in 2016–2017 that caused almost 500 outbreaks and resulted in the culling of 6.8 million birds (Guinat, Nicolas et al., 2018). Following confirmation, all ducks from the infected farm were culled on 6 December 2020 and strict control measures were implemented, including movement restrictions and the establishment of 3 and 10 km radius protection and surveillance zones, respectively. By fitting a mechanistic model of within‐flock transmission to the daily mortality data of this first infected flock in France, we inferred the date of the first infection and within‐flock transmission parameters, which are key to provide policy support and anticipate further spread.

## 2 MATERIALS AND METHODS

The first outbreak occurred in an outdoor breeding mule duck farm (i.e., specialized in the first duck‐production stage of the foie‐gras industry) of 6400 ducks, located in the commune of Bénesse‐Maremne (southwest France). The farm produces 12‐week‐old mule ducks in an all‐in/all‐out production system (i.e., all the ducks enter and leave the farm at the same time). The ducks are dispatched in two adjacent production units, each containing a barn and an outdoor run. The outdoor runs are separated by a single fence that do not prevent ducks from the two runs to have direct contact and, occasionally, even to move from one run to the other. On reaching 12 weeks of age, the ducks are sold to fattening farms where they are raised for 12 days before being sent to slaughter. At the time of the outbreak, ducks were 9‐weeks old. Data on daily duck mortality for the 30 days prior to the day of confirmation were obtained from the farm log book and confirmed by interviewing the farmer. Up to 3 December 2020, daily mortality was stable and low ranging between zero and eight dead ducks. Subsequently, it increased to 40 dead ducks on 4 December (the date when suspicion was reported to veterinary services) and 250 on 5 December. More than 300 ducks were found dead on the morning of 6 December 2020, the day when the flock was culled.

To infer transmission parameters from mortality data, we adapted a modelling framework that was developed previously for African swine fever in the Russian Federation (Guinat et al., 2018). Briefly, the within‐flock transmission of the virus was modelled using a stochastic SEIR epidemic model in which the duck population was divided into four classes: susceptible (S), exposed (i.e., infected but not yet infectious, E), infectious (I) and recovered (R). At the end of their infectious period ducks could either die or survive the infection to recover, with the probability of a duck dying given by the case fatality parameter. We considered a single homogeneously mixing population of ducks since the mortality data were only available for the whole farm, corresponding to the deaths from the two adjacent fields together. The force of infection in the model was given by

$$\lambda \left(t\right)=\beta \frac{I(t)}{N(t)}$$

where β is the transmission rate, I(t) is the number of infectious ducks at time *t* and N(t) is the total number of live ducks at time *t*. To allow for more realistic durations of the latent and infectious periods, we assumed that they follow gamma distributions with mean *m_E_* and *m_I_* and shape parameters *s_E_* and *s_I_*, respectively (Wearing et al., 2005). This means the probability of an individual host completing its latent or infectious period depends on the length of time already spent in the respective classes. The basic reproduction number (*R*_0_), defined as the average number of secondary infections caused by an infectious duck in a totally susceptible flock (Keeling & Rohani, 2007 ), was approximated by $R_{0}=\;\beta \times m_{I}$.

Assuming the outbreak started with a single infected duck, the model was initialized by introducing one duck into the first exposed compartment at the date of virus introduction. However, it is possible that more than one duck became infected at the same time, for example following viral contamination of the field by wild birds. Consequently, we also estimated model parameters assuming five infected ducks at the date of the first infection to check if it had any impact on the model outputs.

The transmission rate, the parameters for the latent and infectious period distributions and the date of virus introduction were estimated by fitting the model to the mortality data using an approximate Bayesian computation sequential Monte Carlo algorithm (Guinat et al., 2018; Toni et al., 2009 ). The summary statistic, which was used as the goodness‐of‐fit metric, was the residual sum of the squared difference between observed and simulated daily mortalities. Because the flock was culled on 6 December, the number of dead ducks observed on the morning of that day (300) did not represent a full day's mortality and, hence, was not used for fitting the model. As shown in Table 1, the transmission rate and the mean durations of the latent and infectious periods were assigned informative gamma priors, calibrated from the literature (Hobbelen et al., 2020; Koeijer et al., 2017 ). In the absence of relevant information, the shape parameters of the latent and infectious periods were given uniform distributions between 0 and 5 and between 0 and 20, respectively. Transmission experiments have been carried out on mule ducks in a controlled environment at the French national reference laboratory for avian influenza using a H5N8 strain isolated during the 2016–2017 epidemic. At the end of the experiment, 39 out of the 56 ducks (70%) had died of the disease, while the remaining ducks survived (Scoizec, personal communication). Consequently, the case fatality parameter was given a beta prior with parameters 40 and 18. The time of virus introduction was given a uniform prior with a range from 15 days prior to the first day mortality data was available to the day of suspicion (day 17). The inference algorithm assumed that all priors were independent from each other. The convergence of the algorithm was assessed by checking the trace plots of all monitored parameters (Supporting Information Figure S1). To explore the sensitivity to the prior distributions, the parameter inference was also conducted after having changed the informative prior distributions of *β*, *m_E_* and *m_I_* to uniform distributions with biologically realistic ranges (Figure 2).

**TABLE 1:** Transmission parameters for highly pathogenic avian influenza virus (H5N8) estimated using mortality data from the index farm of the epidemic that occurred in France in December 2020

Parameter	Description	Prior distribution	Reference	Posterior median	95% credible interval
*t*_0_	Day of introduction	Uniform (−30,17)	–	12.5	11.2−13.9
*Β*	Transmission rate (day^−1^)	Gamma (mean = 1.5; shape = 1.5)	(Koeijer et al., 2017)	4.1	2.8−5.8
*m_E_*	Mean latent period (day)	Gamma (mean = 1; shape = 2)	(Hobbelen et al., 2020)	0.17	0.03−0.38
*s_E_*	Latent period shape	Uniform (0,5)	–	2.4	0.2−4.8
*m_I_*	Mean infectious period (day)	Gamma (mean = 5; shape = 10)	(Hobbelen et al., 2020)	4.3	2.8−5.7
*s_I_*	Infectious period shape	Uniform (0,20)	–	11.4	2.5−19.2
case fatality	Probability of dying of disease	Beta (40,18)	Scoizec, personal communication	0.70	0.61−0.78
*R*_0_	Basic reproduction number	–	–	17.5	9.4−29.3

To predict the within‐flock dynamics of the number of susceptible, exposed and infectious ducks, as well as to explore the model's ability to capture the observed mortality, 1000 replicates of the model were run, sampling from the joint posterior distributions of the parameters that were obtained using the informative priors for *β*, *m_E_* and *m_I_*. The posterior predicted dynamics were summarized with the 5th, 25th, 50th, 75th and 95th percentiles for the number of birds in each class each day. Running more than 1000 simulations did not change the intervals, so this number of simulations was deemed sufficient.

## 3 RESULTS AND DISCUSSION

**FIGURE 1:**
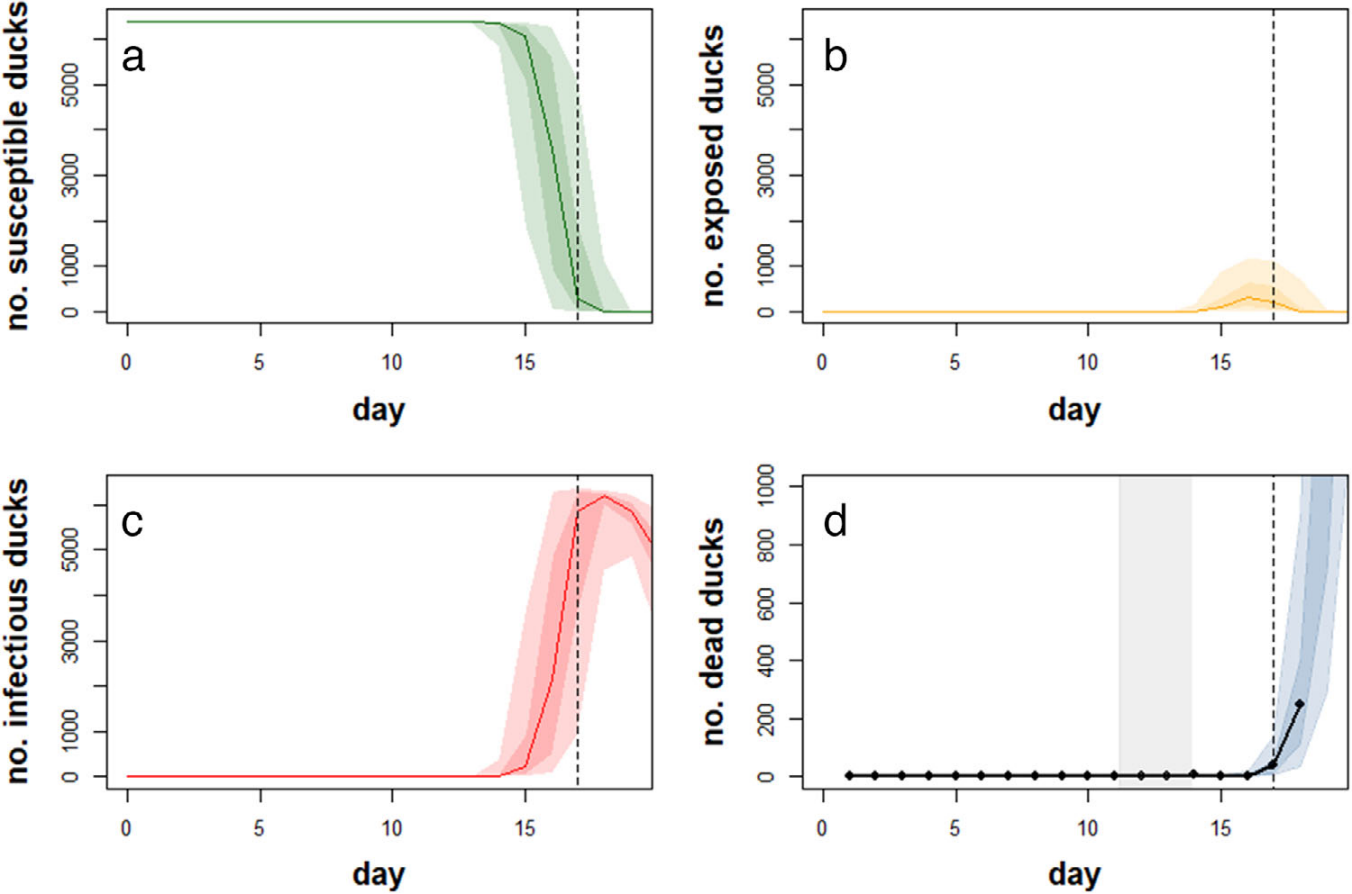
Reconstructed within‐flock dynamics of highly pathogenic avian influenza (H5N8). Panels (a–d) present the evolution of the number of susceptible, exposed, infectious and dead ducks over time with day 0 being the day the flock was culled (6 December 2020). Predicted dynamics are shown as the median (solid lines) and the 50 and 90% posterior prediction intervals (colour shaded areas). Results are based on 1000 replicates of the model sampling from the joint posterior distribution assuming informative priors for all parameters. The grey shaded area indicates the 95% credible interval for the time of the first infection. The vertical dashed line represents the day the suspicion was reported (day 17 = 5 December 2020). In panel (d), the black dots and solid line on panel d represent the observed daily mortality

The predicted within‐flock dynamics of HPAI (H5N8) are illustrated in Figure 1. The model adequately captured the trend in mortality, with the observed daily mortality lying close to the centre of the 50% prediction interval (PI), as shown in Figure 1d). Furthermore, the median number of infectious ducks on the day before suspicion (i.e., at day 16 in Figure 1c) was 2161 (50% PI: 520−4880), the 50% PI corresponding to between 8% and 76% of the total number of ducks in the farm.

The observed mortality data support that the first duck became infected at some time between the 29 November (day 11 in Figure 1) and the 2 December (day 14), that is, between three to six days before HPAI (H5N8) was suspected in the farm (day 17). If we assumed that five ducks became infected at the date of the first infection following an environmental contamination by infected wild birds, these initial infections were likely to have occurred one day later. As summarized in Table 1, the within‐flock transmission rate (β) was estimated to be 4.1 day^−1^ (95% credible interval (CI): 2.8−5.8) and the mean duration of the infectious period (*m_I_*) was estimated to be 4.3 days (95% CI: 2.8−5.7), leading to an *R*_0_ of 17.5 (95% CI: 9.4−29.3). On average, ducks became infectious 0.17 days (95% CI: 0.03−0.38), that is 4.1 h (95% CI: 0.7−9.1) after infection.

**FIGURE 2:**
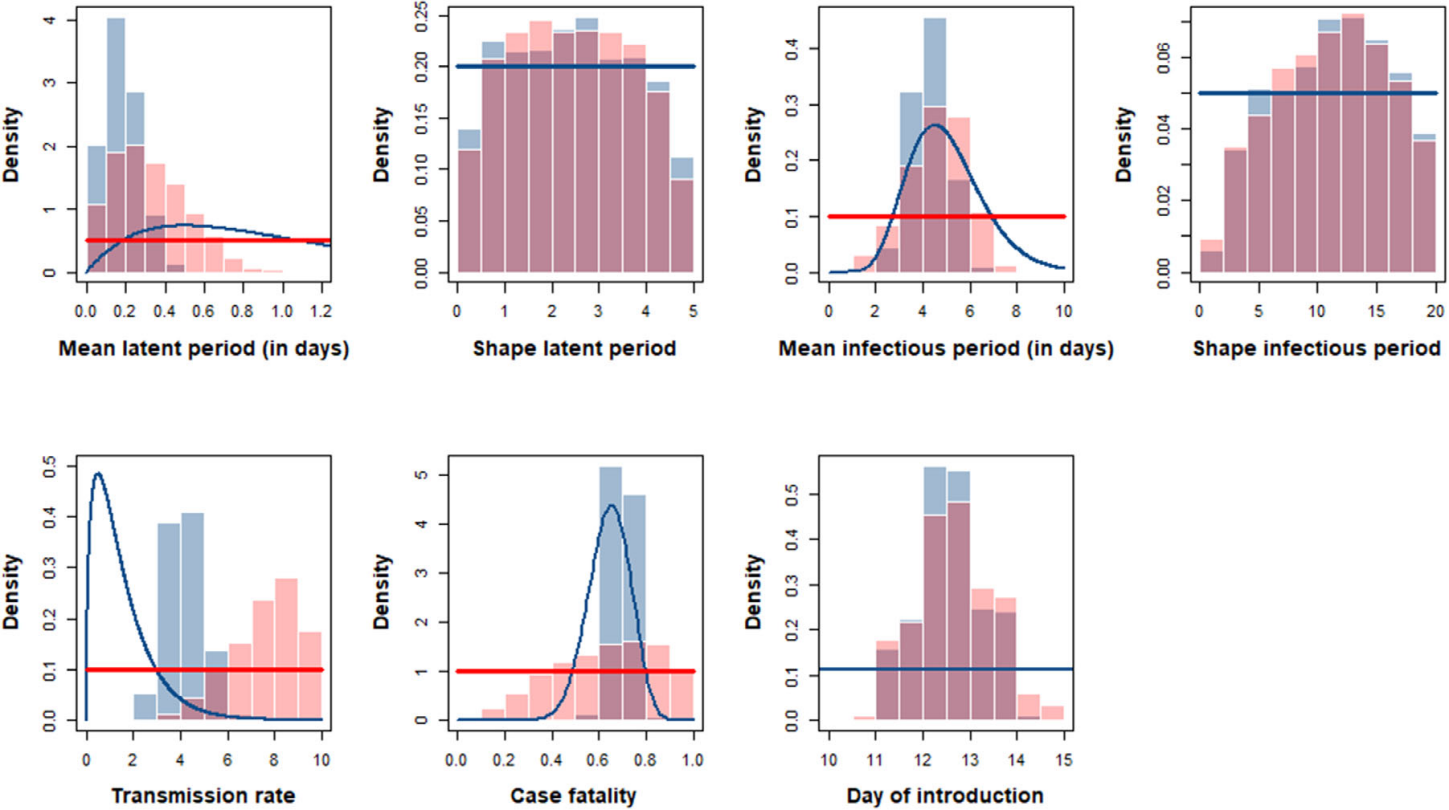
Posterior (histograms) and prior (solid lines) distributions for the model parameters using informative priors (blue) or non‐informative priors (red). Only non‐informative prior distributions were used for the day of introduction and for the shape parameters of the latent and infectious period distributions, and hence, only a single prior appears on the figure

When using informative priors, the posterior and prior distributions were different for the mean latent period, the mean infectious period, the transmission rate and the day of introduction, suggesting a substantial contribution of the data to their estimates (Figure 2). However, the prior and posterior distributions were not substantially different for the shape parameters of the latent and infectious period distributions or for the case fatality, indicating a lack of information in the data for these parameters. The choice of prior distributions had a substantial influence on the estimates for the transmission rate and the case fatality, while it had only a limited impact on the estimates for the mean duration of the latent and infectious periods (Figure 2). If we assumed that five ducks (instead of one) became infected at the time of the first infection, the posterior distributions for the transmission parameters did not change (Supporting Information Figure S2).

Despite avian influenza being one of the most devastating diseases in poultry, few studies (Gonzales et al., 2012; Hobbelen et al., 2020 ) have estimated within‐flock transmission parameters using data from real outbreaks. Our estimates of the transmission rate (4.1; 95 CI%: 2.8−5.8) and the mean length of infectious period (4.3 days; 95% CI: 2.8−5.7) led to an estimate of *R*_0_ of 17.5 (95% CI: 9.4−29.3). These estimates are consistent with the fast increasing mortality observed in the field as well as with those from a modelling study performed on H5N8 clade 2.3.4.4 outbreaks in The Netherlands in 2016 (Hobbelen et al., 2020). The estimated delay between infection and detection is also in accordance with the results from a mechanistic model of between‐farm transmission of HPAI (H5N8) during the 2016–2017 epidemics in France (Andronico et al., 2019). Because the model assumed only one population while the ducks were distributed in two outdoor production units, the estimated value for the transmission rate could underestimate the true value. However, ducks from the two different production units could have direct contact on the outdoor run through the fence. Furthermore, the spread of the virus was extremely fast. Accordingly, we expect that the assumption of a single population will have only a limited impact on the estimates.

Estimates of within‐flock transmission parameters bring valuable insights for outbreak response. Our results suggest that, due to a high transmission rate and a relatively long infectious period, the within‐flock prevalence of infectious ducks was already extremely high the day before unusual mortality was observed in the farm. This finding emphasizes the substantial latent threat this virus could pose to other poultry farms and to neighbouring wild birds, despite rapid and efficient clinical surveillance. This calls for the implementation of strengthened preventive measures in outdoor poultry production during high‐risk periods. The accurate estimation of the time of the first infection should also help define the time window on which to focus epidemiological investigations and control efforts on infected farms (Hobbelen et al., 2020). The methods used in this study could be applied to future outbreaks and the results made available to veterinary services within a few hours. The results obtained in this short study now need to be consolidated and validated using mortality data from other infected farms that were subsequently suspected based on an increase in duck mortality.

In conclusion, our study illustrates how mechanistic models can provide rapid relevant insights to contribute to the management of infectious disease outbreaks of farmed animals.

## CONFLICT OF INTEREST

The authors declare no conflict of interest.

## ETHICAL APPROVAL

Because the mortality data used to fit the model were collated by the farmer as part of the farm's routine management procedures, formal approval from an Ethics Committee was not a requirement for this study.

## Supporting information

SUPPORTING INFORMATION

## Data Availability

The mortality data that support the findings of this study are available as Supporting Information. The code implementing the ABC‐SMC algorithm are available online at: https://github.com/SimonGubbins/ABC‐SMC_MortalityData
