# Supplementary material for: Inferring within‐flock transmission dynamics of highly pathogenic avian influenza H5N8 virus in France, 2020
Source: Transbound Emerg Dis. 2021 Jul 27;68(6):3151–5. doi: 10.1111/tbed.14202 (PMC9291964; doi:10.1111/tbed.14202)
Supplement: Supplementary file 1 — SUPPORTING INFORMATION [file TBED-68-3151-s001.docx]

Supplementary information

**Inferring within-flock transmission dynamics of highly pathogenic avian influenza H5N8 virus in France, 2020**

**Timothée Vergne^1^, Simon Gubbins^2^, Claire Guinat^3,4^, Billy Bauzile^1^, Mattias Delpont^1^, Debapriyo Chakraborty^1^, Hugo Gruson^5^, Benjamin Roche^5,6,7^, Mathieu Andraud^8^, Mathilde Paul^1^, Jean-Luc Guérin^1^**

*^1^ UMR ENVT-INRAE IHAP, National Veterinary School of Toulouse, Toulouse, France*

*^2^ The Pirbright Institute, Pirbright, Surrey, United Kingdom*

*^3^ Department of Biosystems Science and Engineering, ETH Zurich, Basel, Switzerland*

*^4^ Swiss Institute of Bioinormatics (SIB), Switzerland*

*^5^ UMR IRD-CNRS-UM2 MIVEGEC, Institut de Recherche pour le développement, Montpellier, France*

*^6^ IRD, Sorbonne Université, UMMISCO, Bondy, France*

*^7^ Facultad de Medicina Veterinaria y Zootecnia, Universidad Nacional Autónoma de México (UNAM), Ciudad de México, México*

*^8^ ANSES, Ploufragan-Plouzané-Niort Laboratory, Epidemiology, Health and Welfare research unit, Ploufragan, France*

*Table S1: Daily mortality data supporting the findings of this study*

| **Day** | **Nb of dead ducks** |
| --- | --- |
| 1 | 0 |
| 2 | 0 |
| 3 | 1 |
| 4 | 1 |
| 5 | 2 |
| 6 | 3 |
| 7 | 1 |
| 8 | 3 |
| 9 | 2 |
| 10 | 1 |
| 11 | 5 |
| 12 | 0 |
| 13 | 0 |
| 14 | 8 |
| 15 | 2 |
| 16 | 2 |
| 17 | 40 |
| 18 | 250 |


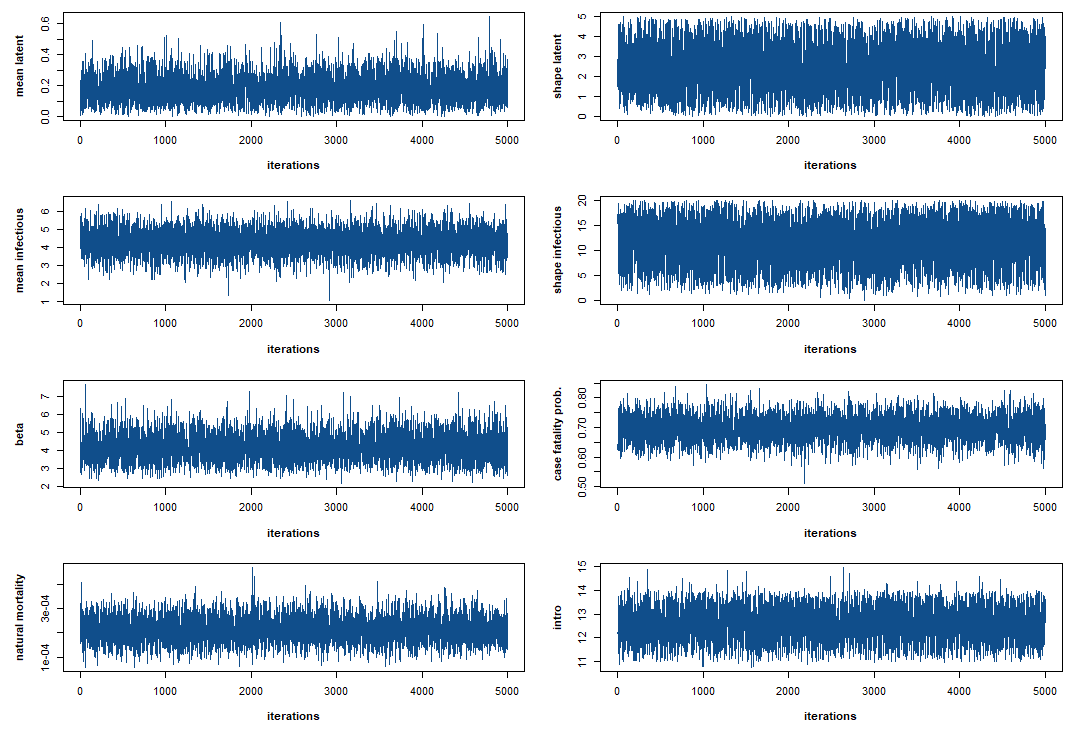


*Figure S1: Trace plot of the monitored parameters for the last round of the ABC-SMC algorithm.*


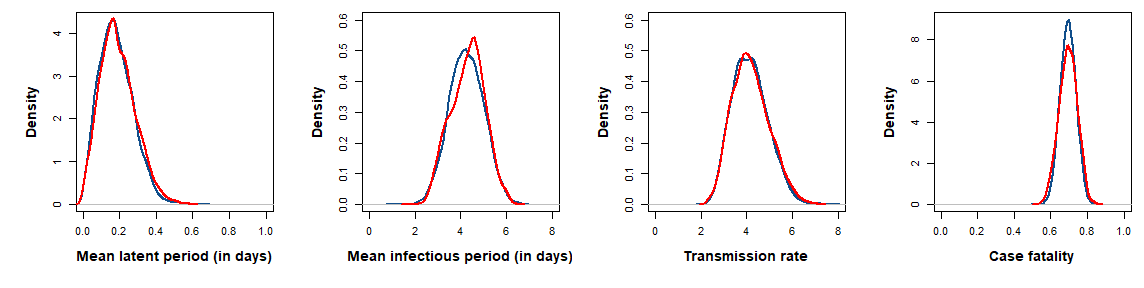


*Figure S2: Impact of initial conditions on the posterior distributions for the transmission parameters. The red and blue lines correspond to the posterior distributions assuming that one or five ducks became infected at the time of the first infection, respectively, assuming informative priors for all parameters.*
